# Uptake and Effectiveness of Outpatient vs. Residential Cardiac Rehabilitation After Myocardial Infarction: A Nationwide Analysis

**DOI:** 10.5334/gh.1470

**Published:** 2025-09-12

**Authors:** Borut Jug, Zlatko Fras, Tjaša Furlan, Marko Novaković, Jerneja Tasič, Mitja Lainščak, Jerneja Farkaš, Dalibor Gavrić, Irena Ograjenšek, Petra Došenović Bonča

**Affiliations:** 1 Centre for Preventive Cardiology, Department of Vascular Medicine, University Medical Centre Ljubljana, Slovenia; 2 Faculty of Medicine, University of Ljubljana, Slovenia; 3 General Hospital Trbovlje, Slovenia; 4 National Institute of Public Health, Slovenia; 5 General Hospital Murska Sobota, Slovenia; 6 Health Insurance Institute of Slovenia, Slovenia; 7 Faculty of Economics and Business, University of Ljubljana, Slovenia

**Keywords:** Cardiac rehabilitation, center-based, Cardiac rehabilitation, short-term, Myocardial infarction, Comparative effectiveness research, Outcomes research, Propensity score, Cost-effectiveness analysis

## Abstract

**Aims:**

To estimate the participation in, and the comparative effectiveness of, short-term residential and comprehensive outpatient cardiac rehabilitation (CR), after the latter was introduced in Slovenia by establishing dedicated regional CR centers.

**Methods:**

We extracted and analyzed data on all patients hospitalized for myocardial infarction in Slovenia (*n* = 15,639), focusing on CR participation – either comprehensive outpatient (introduced in 2017) or short-term residential (available throughout the study period 2015–2021). Impact on nation-wide CR participation rates was assessed by interrupted time series analysis; impact on patient-level outcomes (all-cause mortality and cardiovascular hospitalizations) was assessed using Kaplan Meier estimators and ‘doubly robust’ Cox regression with propensity score-derived inverse probability of treatment weighting.

**Results:**

Of the 11,815 eligible patients (event-free after 180-day landmark), 3819 (32.3%) attended CR. Nation-wide CR participation rates increased both in level (9.7%, 95% CI 6.3–3.1) and in trend (0.41% per month, 95% CI 0.22–0.60) after outpatient CR was introduced in 2017. After propensity score-based adjustment, participation in either CR was associated with lower event rates (12.8%, 17.2%, and 21.0% at 3-year follow-up for outpatient, residential, and no CR, respectively; *p* < 0.001). Risk reductions were significant for composite outcomes (outpatient: HR 0.58, 95% CI 0.47–0.70; residential: HR 0.79, 95% CI 0.68–0.93) and all-cause mortality (outpatient: HR 0.56, 95% CI 0.38–0.83; residential: HR 0.59, 95% CI 0.45–0.77), whereas the risk reduction for cardiovascular hospitalizations was only significant for outpatient CR (HR 0.60, 95% CI 0.48–0.74). The incremental cost-effectiveness ratio per life-year gained was €6421 and €7381 for outpatient and residential CR, respectively.

**Conclusions:**

Participation in either CR improves outcomes after myocardial infarction, but comprehensive outpatient CR conveys superior risk reductions, primarily through reduced cardiovascular hospitalizations.

**Lay Summary:**

- Our study highlights the importance of expanding cardiac rehabilitation services (by setting up dedicated regional comprehensive outpatient centers) and provides new evidence on improved outcomes in patients after myocardial infarction, who undergo cardiac rehabilitation. While previous studies have demonstrated the efficacy and effectiveness of cardiac rehabilitation, ours is the first to compare two distinctive cardiac rehabilitation modalities – comprehensive outpatient (introduced in 2017) and short-term residential (available throughout the study period 2015–2021).
- In our nationally representative population of patients after myocardial infarction (*n* = 15,639), participation in cardiac rehabilitation increased both in level (by ~10%) and in trend (by ~0.4% per month) after dedicated cardiac rehabilitation centers were established. Participation in either comprehensive outpatient or short-term residential cardiac rehabilitation was associated with a significant 42% and 21% risk reduction in the primary outcome (death or cardiovascular hospitalization) respectively, after propensity score-based adjustment. Mortality was also reduced (by 46% and 41%, respectively), whereas the risk reduction for hospitalization was only significant in patients undergoing comprehensive cardiac rehabilitation (by 60%).
- Participation in either cardiac rehabilitation program improves cardiovascular outcomes, but comprehensive outpatient cardiac rehabilitation yields superior risk reductions, primarily through reduced cardiovascular hospitalizations.

**Key Learning Points:**

**What is already known?**

- Cardiac rehabilitation improves outcomes in patients with coronary artery disease.
- Despite its established efficacy, cardiac rehabilitation participation remains suboptimal. Improving access to cardiac rehabilitation through establishing dedicated regional centers may improve participation, but also crowd-out existing options of cardiac rehabilitation.
- The comparative effectiveness of different cardiac rehabilitation modalities (e.g., comprehensive outpatient versus short-term residential cardiac rehabilitation) remains understudied.

**What does this study add?**

- Expanding cardiac rehabilitation services (by setting up dedicated regional comprehensive outpatient centers) significantly improves participation in cardiac rehabilitation after myocardial infarction.
- Participation in either comprehensive outpatient or short-term residential cardiac rehabilitation after myocardial infarction is associated with improved outcomes (i.e., a significant 42% and 21% risk reduction in death or cardiovascular hospitalization, respectively).
- Comprehensive outpatient cardiac rehabilitation yields superior risk reductions primarily through reduced cardiovascular hospitalizations.

## Introduction

Cardiac rehabilitation (CR) is a complex intervention aimed at improving cardiovascular health through several core components, including exercise training, risk factor control, secondary prevention, and psychosocial support (1). For patients after myocardial infarction, participation in CR is a class I recommendation and a measure of quality of care (2). While the delivery of CR around the globe may vary (3), exercise-based CR should ideally be provided through multidisciplinary and comprehensive programs, encompassing several core components and at least 36 supervised sessions over several months (4,5). Center-based outpatient comprehensive CR programs not only provide such structure and duration but can also accommodate enough evidence-based core interventions to elicit improvements in cardiovascular health (6,7). Other modalities, such as short-term residential CR (wherein the patients reside in a dedicated CR facility for a short time, e.g., for 2–4 weeks), primarily focus on patients’ recovery after a major cardiovascular event. Short-term residential CR programs – while limited in duration and based on comparatively fewer outcomes research studies (8,9) – still provide CR benefits to patients after myocardial infarction and thus remain popular in continental Europe, especially in German-speaking countries (10).

In Slovenia, prior to 2017, CR was underutilized even in academic cardiovascular centers (11), and the options for CR were limited to short-term residential programs (12,13). To expand CR content and provision, key stakeholders (including the Slovenian Society of Cardiology, the Health Insurance Institute of Slovenia, the National Institute of Public Health, and the Slovenian Forum for Cardiovascular Disease Prevention) proposed the set-up of comprehensive outpatient CR centers affiliated with each regional hospital in the country (14). Starting in 2017, seven regional centers were established to provide comprehensive multidisciplinary outpatient CR. The introduction of regional comprehensive outpatient CR programs in Slovenia provides a unique opportunity to assess the impact on nation-wide CR participation and to estimate the real-life comparative effectiveness of participation in either comprehensive outpatient or short-term residential CR.

In terms of CR participation, CR remains underutilized worldwide (15). While some studies have observed favorable secular trends in CR utilization (16), the impact of specific interventions – such as introducing new CR delivery services – remains elusive. In terms of real-life effectiveness, observational evidence from population-based cohorts may complement information derived from clinical trials; while the latter provides robust causality assumptions on CR efficacy (internal validity), the former expands the generalizability of CR effectiveness to real-life populations (external validity). Observational analyses drawing from large healthcare datasets have previously shown that CR participation is associated with survival benefits in patients with coronary artery disease, with reported risk reductions ranging from 33% to over 50% for all-cause mortality (8,10,16,17,18,19,20,21,22,23,24,25). Differences in observed survival benefits may be attributed to differences in study eras, referral indications, CR modalities, study populations, and countries. Importantly, previous research was limited by focusing on participation in any CR without specific comparisons between two or more available modalities (such as comprehensive outpatient CR and short-term residential CR).

The aim of the present study was twofold: (1) to assess the impact of establishing regional comprehensive outpatient CR centers on CR participation rates, and (2) to estimate the comparative effectiveness of either comprehensive outpatient or short-term residential CR participation in improving outcomes after myocardial infarction.

## Methods

### Study population, data sources, and analytic cohort

This was a longitudinal observational analysis with a retrospective design of all patients hospitalized for myocardial infarction (index diagnosis, ICD codes I21.x) in Slovenia between January 1, 2015 and January 1, 2021 (nationwide patient population), focusing on patients undergoing CR (index intervention). The study protocol was approved by the Medical Ethics Committee of the Republic of Slovenia (KME No. 0120-223/2021/11), and the analysis was conducted in compliance with the Slovenian and European Union regulations and legislative frameworks.

Data were drawn from available databases at the Health Insurance Institute of Slovenia, which provides universal healthcare coverage for the whole country (~2.1 million inhabitants/universally insured) and has been monitoring healthcare system activity through centralized electronic data collection (with complete nationwide data collection since 2015). Data were obtained by merging (1) the National Hospital Management Database (patients with index diagnosis), (2) National Medicines and Cardiac Rehabilitation Reimbursement claims (index intervention), and (3) the Central Population Registry, using unique patient identifiers of the Health Insurance Institute. After linkage (DG), datasets were deidentified for research analysis purposes and restricted to the core research group (BJ and PDB). Patients without national Health Insurance Institute of Slovenia coverage (e.g., tourists and non-residents) could not be included.

### Description of cardiac rehabilitation

Short-term residential CR was the predominant CR modality prior to 2017 and remained available throughout the study period 2015–2021; it involves a relatively short 2-week duration with patients residing in-center. Comprehensive outpatient CR programs were established in 2017 and provide CR over a 3-month duration in the outpatient setting (See Supplementary Table 1 for a comparative description of CR modalities).

**Figure 1:**
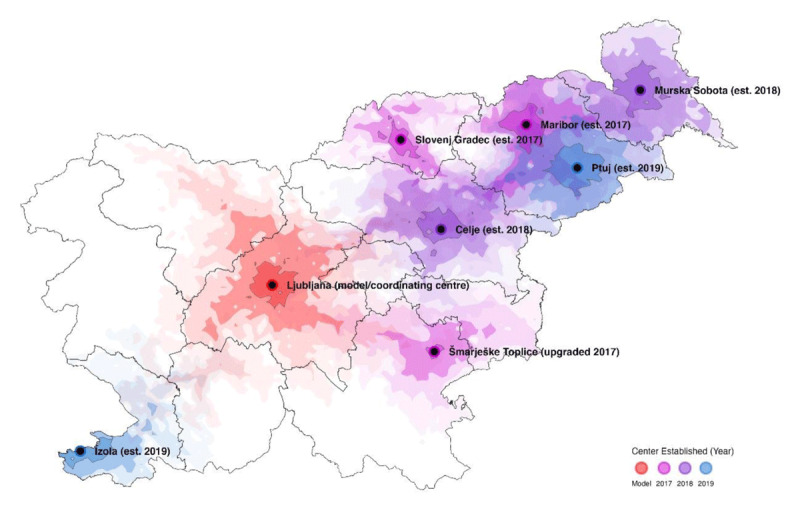
Map of comprehensive outpatient cardiac rehabilitation centers (color-coded for timeline of set-up and isochrones representing distance in 15-, 30-, 45- and 60-min driving times).

The implementation of hospital-affiliated center-based comprehensive outpatient CR programs started in January 2017, and comprised dedicated facility set-up and personnel training, introduction of referral pathways to CR (automated referral for CR intake visit for all hospitalized patients with myocardial infarction, with recommended intake within 30 days following hospital discharge). Set-up was completed within a 6-month time frame under the supervision of a coordinating team (Centre for Preventive Cardiology, University Medical Centre Ljubljana). Maximal CR capacity was set to 150 patients/center/year (and increased to 300 patients/center/year at the coordinating center). Minimal staffing requirements were: cardiologist, nurse, physical/exercise therapist, and administrative support; center affiliation with regional hospital was mandatory (i.e., access to emergency services, laboratory, hospitalization, mental health services, advanced/clinical dietary services, and occupational medicine services). Full reimbursement was contingent on completion of 36 exercise sessions but was mutually exclusive for participation in residential short-term CR (i.e., reimbursement for only one CR program). First patient enrolment started on June 5, 2017. By 2020, seven fully functional and accredited CR programs were established at seven hospitals (out of the 14 in the country) and at one residential CR center (Figure 1).

### Outcomes and confounders

Participation in CR was defined if a claim was filed for at least one CR session (beyond intake visit); treatment status (no CR, short-term residential CR, or comprehensive outpatient CR) was defined by participation in specific CR program between index event hospitalization (myocardial infarction) and a 180-day landmark, in line with previous observational studies (16,19,21). For the nationwide CR participation analysis, CR participation rate (i.e., uptake) was defined as the proportion of patients after myocardial infarction who participated in any CR program.

For the patient-level outcomes analysis, focusing on the estimation of CR effectiveness, the primary outcome of interest was time to first composite outcome, defined as death from any cause or cardiovascular hospitalization (i.e., non-fatal myocardial infarction [ICD-10 diagnosis I21.x], other non-fatal ischemic heart disease [I20.x, I22.x-I25.x], non-fatal stroke/cerebrovascular disease [I63.x, I65.x, I66.x], heart failure [I50.x], or peripheral artery/aortic disease [I70.x, I71.x]). Secondary outcomes of interest were time to death from any cause and time to first cardiovascular hospitalization. Other outcomes were all-cause mortality and cardiovascular hospitalization at 6 months, and emergency department visit and retention of key secondary preventive medication at 12 months (i.e., between landmark 180 days and 365 days from index hospitalization discharge).

Co-morbidities were defined by recorded entries in the Hospital Management Database and/or by medication claims for specific conditions (e.g., antidiabetic medication excluding SGLT2 for diabetes mellitus, antidepressants for depression, dementia-specific medications for dementia; inhalation medication for pulmonary obstructive syndromes, anti-ischemic medication for residual myocardial ischemia), based on the ATC classification. Key secondary preventive medications were defined as: intensive antithrombotic therapy (defined as either dual antiplatelet therapy [aspirin plus P2Y12 antagonist] or single antiplatelet plus oral anticoagulation therapy), lipid-lowering therapy (defined as statin therapy, ezetimibe, PCSK9 inhibitor, or combination thereof), beta blocking therapy, and renin-angiotensin [RAAS] inhibitor therapy (defined as angiotensin converting enzyme [ACE] inhibitor or angiotensin receptor blocker [ARB]).

## Statistical Analysis

### Descriptive statistics

Summary descriptive statistics are expressed as medians (and interquartile ranges, IQR) for continuous variables and as numbers (and proportions) for categorical variables. Exploratory comparisons between independent groups (e.g., baseline characteristics) were assessed using the Kruskal–Wallis test (for continuous variables) or the χ^2^ test (for categorical variables). Statistical significance was set at two-tailed *p* < 0.05. Statistical analyses were performed with R/RStudio ver. 2024.04.2+764, and using the WeightIt and Cobalt packages (26,27).

#### Nation-wide CR participation rates – interrupted time series analysis

We hypothesized that the introduction of the comprehensive outpatient CR programs would increase the level and trend for overall CR participation rates; we also considered the external shock of the coronavirus-19 (COVID-19) pandemic outbreak, which we assumed would cause a reversal in level and in trend of CR participation. Thus, data were fitted to segmented regression models for interrupted time-series (28). Two breakpoints were defined prior to analysis – June 2017 (introduction of the comprehensive outpatient CR programs with first patient enrolment) and March 2020 (COVID-19 pandemic outbreak based on epidemiological data, enacted government responses, and COVID-19 stringency index for Slovenia) (29). Model assumptions were assessed with correlograms (autocorrelation and partial autocorrelation function) and residual plots, and refitted/adjusted as appropriate (e.g., differencing for stationarity, dummy variables for seasonality, and/or model adjustment for autoregressive orders). We performed pre-defined subgroup analyses for: STEMI versus NSTEMI, men versus women, age <65 years versus age ≥65 years, and university-affiliated versus general hospital. Sensitivity analyses were performed by extending the transition period (intervention roll-out) to Jan.–Jun. 2017.

#### Patient-level outcomes – survival analysis with propensity score-derived inverse probability of treatment weights

Patient-level characteristics were used to estimate propensity scores (the probability of treatment/CR assignment conditional on observed time-invariant baseline covariates at discharge from index hospitalization) and weights (the inverse probability of receiving the treatment that was actually received). The following baseline covariates were a priori defined: age (in years), sex (male/female), type of myocardial infarction (STEMI/NSTEMI), relevant risk-associated recorded concomitant diagnoses (diabetes mellitus, arterial hypertension, atrial fibrillation, heart failure, residual myocardial ischemia, dementia, cancer, chronic kidney disease, chronic obstructive pulmonary disease [COPD] or asthma), hospital episode characteristics (length of stay ≥5 days, disease-related group [DRG] intensity as well as the total number of coded diagnoses and the total number of coded procedures, capturing all diagnoses and procedures coded during the hospital episode, including those not captured by DRG groupers), discharge hospital characteristics (university/general hospital; hospital with/without affiliated comprehensive CR program), socioeconomic status (obtained using postal code information for patients’ residence, and categorized into quartiles), and key secondary preventive medication at discharge (intensive antithrombotic therapy, lipid-lowering therapy, β-blockers, RAAS inhibitors). Covariates were defined by recorded entries in the Hospital Management Database and/or by reimbursement claims, with variables either coded or non-coded (i.e., no missing variables in the analytic dataset).

The target estimate was average treatment of the treated, focusing on participation in comprehensive outpatient CR. Propensity scores for multiple treatments (i.e., no CR participation, short-term residential CR participation, or comprehensive outpatient CR participation) were estimated using multinomial logistic regression, generalized boosted modeling, and covariate balancing propensity score (30); the latter was chosen based on covariate balancing diagnostics (standardized mean differences [SMDs] and Kolmogorov–Smirnov statistics).

Treatment effects of CR on time-to-event outcomes were assessed by fitting a ‘double-robust’ Cox proportional hazard regression model (i.e., weighted for inverse probability of treatment *and* adjusted for all covariates, with robust standard errors estimation) and by adjusted Kaplan–Meyer curves (31). Cox analysis results are reported with hazard ratios (HR) and 95% confidence intervals (95% CI). Survival time (in days) was defined by a minimum (landmark) 180 days/6 months and a maximum follow-up time of 3 years (limited by follow-up time for patients in comprehensive outpatient CR, with earliest enrolment in June 2017, landmark throughout January 1, 2018, and maximum follow-up by January 2021). The proportional hazards assumption was assessed by Schoenfeld residuals.

Sensitivity analyses were performed by changing the intervention window time (i.e., narrowing from 180 to 90 days), by estimating inverse probability of treatment derived using multinomial logistic regression and generalized boosting model-derived propensity scores, by weighted Cox regression modeling without covariate adjustment, and by standard conditional Cox regression modeling (including all covariates used for propensity score estimation) without weighting. In addition, we performed logistic regression for exploratory analysis for CR participation, CR completion (all 36 sessions of outpatient or all 14 days of residential CR, respectively), and binary outcomes (i.e., mortality, cardiovascular hospitalizations, emergency department visits, and key secondary preventive medication retention) at 12 months follow-up.

### Cost-effectiveness analysis

A cost-effectiveness analysis (i.e., incremental cost per year-lives gained) was conducted from the payer’s perspective in the Slovenian healthcare system. Survival data were analyzed using restricted mean survival time to a 3-year time horizon; life-years gained were estimated from differences in restricted mean survival time between each rehabilitation group and control. Costs per patient (overall healthcare costs, i.e., rehabilitation and further hospital care, emergency department/outpatient visit, and medication reimbursement claims, adjusted for inflation and discounted at an annual rate of 4%) were used to calculate weighted mean costs (using propensity score-derived inverse probability of treatment weights) for each group. Probabilistic sensitivity analysis was performed with non-parametric bootstrapping to generate confidence intervals, cost-effectiveness planes, and cost-effectiveness acceptability curves.

## Results

**Figure 2:**
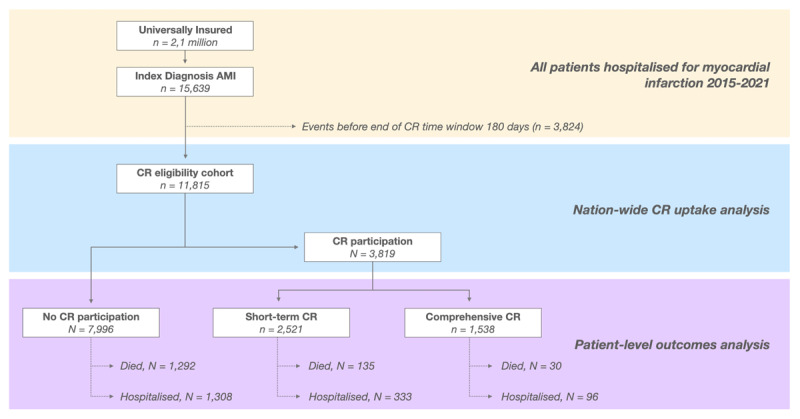
Flow of participants through analysis.

Data on 15,639 patients with a myocardial infarction diagnosis between 2015 and 2021 were extracted; 3824 (24.5%) patients were excluded because of events within the 180-day time window from discharge, yielding 11,815 patients for the analysis (Figure 2). Overall, 3819 (32.3%) patients attended CR (either comprehensive or short-term residential). Median time from discharge to CR was 54 (IQR 25–154) days, with 1414 (37%) patients meeting the recommended 30-day target waiting time for CR enrolment. In the comprehensive outpatient CR group, 723 (47%) patients completed all 36 sessions, in the short-term CR group, 2158 (86%) patients completed 14 days of residential CR.

### Nationwide cardiac rehabilitation participation

**Figure 3:**
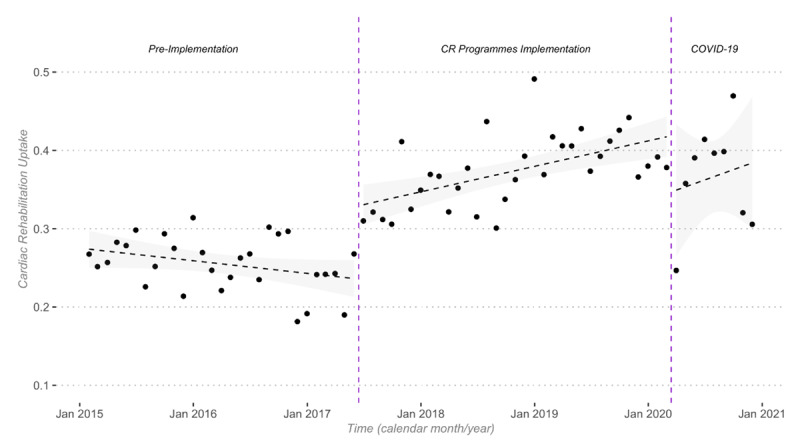
Interrupted time-series of cardiac rehabilitation (CR) participation rates.

CR participation rates increased from 24.8% in 2016 to 40.1% in 2019, and then decreased to 37.4% in 2020 (COVID-19 pandemic). The introduction of outpatient comprehensive CR programs (June 2017) yielded a significant increase in level (9.7%, 95% CI 6.3 to 13.1%, *p* < 0.001) and in trend (0.41% per month, 95% CI 0.22 to 0.6 per month, *p* < 0.001) of CR participation (Figure 3). The COVID-19 pandemic outbreak was associated with a significant decrease in level (–7.2%, 95% CI –13.8 to –0.6%, *p* = 0.038) and a non-significant change in trend (0.17% per month, 95% CI –1.05 to 1.39, *p* = 0.788). Subgroups with the highest overall CR participation rates and the most pronounced impact on CR participation were patients with STEMI (vs. NSTEMI), men (vs. women), aged 65 years or less (vs. ≥65 years), and patients discharged from university hospitals (vs. general hospitals). See Supplementary Table 2.

### Patient-level outcomes

We observed significant differences across all baseline covariates between non-participants, patients undergoing short-term residential CR, and patients undergoing comprehensive outpatient CR (Table 1). Patients undergoing CR, especially comprehensive outpatient CR, were significantly younger, more often men, more likely to have suffered STEMI (as opposed to NSTEMI); had a lower prevalence of recorded diabetes, hypertension, atrial fibrillation, heart failure, cancer, chronic kidney disease, COPD/asthma, depression, and lower total number of co-morbidities; had higher-intensity hospital episodes (higher number of procedures and DRG intensity over a shorter length of stay); were more likely discharged from a university hospital and/or resided in higher level socioeconomic communities; and had higher rates of prescribing intensive antithrombotic therapy, lipid-lowering therapy, β-blockers, and RAAS inhibitors at discharge. Between-group differences, predictors of CR participation, and predictors of CR completion are detailed in Supplementary Tables 3–5.

**Table 1:** Baseline characteristics for non-participants, short-term residential cardiac rehabilitation participants, and comprehensive outpatient cardiac rehabilitation participants.


CHARACTERISTIC	OVERALL	NO CR	SHORT-TERM	COMPREHENSIVE	*p*-VALUE

	*N* = 11815	*N* = 7996	*N* = 2281	*N* = 1538	

Age	66 (57–77)	69 (59–79)	63 (55–71)	58 (52–66)	<0.0001

Sex (male)	7882 (67%)	5030 (63%)	1676 (73%)	1176 (76%)	<0.0001

STEMI	5431 (46%)	3112 (39%)	1410 (62%)	909 (59%)	<0.0001

Diabetes	2631 (22%)	1827 (23%)	564 (25%)	240 (16%)	<0.0001

Arterial hypertension	7245 (61%)	5030 (63%)	1384 (61%)	831 (54%)	<0.0001

Atrial fibrillation	1481 (13%)	1147 (14%)	260 (11%)	74 (4.8%)	<0.0001

Heart failure	2094 (18%)	1366 (17%)	539 (24%)	189 (12%)	<0.0001

Depression	811 (6.9%)	585 (7.3%)	147 (6.4%)	79 (5.1%)	0.0056

Dementia	264 (2.2%)	258 (3.2%)	5 (0.2%)	1 (<0.1%)	<0.0001

Malignancy	358 (3.0%)	285 (3.6%)	51 (2.2%)	22 (1.4%)	<0.0001

Chronic kidney disease	966 (8.2%)	768 (9.6%)	155 (6.8%)	43 (2.8%)	<0.0001

COPD or asthma	1038 (8.8%)	748 (9.4%)	191 (8.4%)	99 (6.4%)	0.0008

Antiplatelet therapy	11212 (95%)	7493 (94%)	2189 (96%)	1530 (99%)	<0.0001

Lipid-lowering therapy	9727 (82%)	6250 (78%)	2018 (88%)	1459 (95%)	<0.0001

RAAS inhibitors	8544 (72%)	5629 (70%)	1714 (75%)	1201 (78%)	<0.0001

β-Blockers	8834 (75%)	5682 (71%)	1871 (82%)	1281 (83%)	<0.0001

Anti-ischemic therapy	2381 (20%)	1660 (21%)	479 (21%)	242 (16%)	<0.0001

Revascularization	10319 (87%)	6731 (84%)	2119 (93%)	1469 (96%)	<0.0001

Number of diagnoses*	4 (2–5)	4 (2–5)	4 (3–6)	3 (2–4)	<0.0001

Number of procedures*	15 (9–19)	13 (7–18)	16 (10–20)	17 (13–20)	<0.0001

Length of stay ≥5 days	6242 (53%)	4098 (51%)	1508 (66%)	636 (41%)	<0.0001

DRG intensity*	3.4 (2.4–3.8)	3.2 (2.3–3.8)	3.4 (2.7–4.2)	3.6 (3.2–3.8)	<0.0001

University hospital	6895 (58%)	4501 (56%)	1318 (58%)	1076 (70%)	<0.0001

Hospital with CR center**	9986 (85%)	6710 (84%)	1844 (81%)	1432 (93%)	<0.0001

Socioeconomic status					<0.0001

High	2555 (22%)	1706 (21%)	410 (18%)	439 (29%)	

Middle-high	2706 (23%)	1961 (25%)	459 (20%)	286 (19%)	

Middle-low	2545 (22%)	1761 (22%)	526 (23%)	258 (17%)	

Low	4009 (34%)	2568 (32%)	886 (39%)	555 (36%)	


CR, cardiac rehabilitation; STEMI, ST elevation myocardial infarction (vs. non-ST elevation myocardial infarction); COPD, chronic obstructive pulmonary disease; RAAS, renin-angiotensin system inhibitors; CABG, coronary artery bypass grafting; PCI, percutaneous coronary intervention. *DRG – disease-related group; reflecting the intensity of hospital episode, including case mix, diagnoses, procedures, and complications. Total number of coded diagnoses and procedures, capturing all diagnoses and procedures coded during the hospital episode, including diagnoses and procedures not captured by DRG intensity. **Hospital providing affiliated cardiac rehabilitation center.

Inverse probability of treatment weighing with propensity scores yielded comparable baseline covariates across groups/pseudo-cohorts (no CR participation, patients undergoing short-term residential CR, and patients undergoing comprehensive outpatient CR; Table 2 and Supplementary Figure 1).

**Table 2:** Baseline characteristics for non-participants, short-term residential cardiac rehabilitation participants, and comprehensive outpatient cardiac rehabilitation participants after weighting.


	OVERALL	NO CR	SHORT-TERM	COMPREHENSIVE	*p*-VALUE	MAX SMD	KOLMOGOROV–SMIRNOV

	*N* = 4609*	*N* = 1534*	*N* = 1537*	*N* = 1538*			

Age	58 (51–66)	58 (50–67)	59 (51–66)	58 (52–66)	0.66	0.0047	0.0478

Sex (male)	76.4%	76.3%	76.5%	76.5%	>0.99	0.0012	0.0012

STEMI	59.1%	59.2%	59.1%	59.1%	>0.99	0.0010	0.0010

Diabetes	15.6%	15.6%	15.6%	15.6%	>0.99	0.0001	0.0001

Arterial hypertension	54.0%	54.1%	54.0%	54.0%	>0.99	0.0003	0.0003

Atrial fibrillation	4.8%	4.8%	4.8%	4.8%	>0.99	0.0004	0.0004

Heart failure	12.3%	12.3%	12.3%	12.3%	>0.99	0.0003	0.0003

Depression	5.1%	5.2%	5.1%	5.1%	>0.99	0.0003	0.0003

Dementia	0.1%	0.1%	0.1%	0.1%	>0.99	0.0000	0.0000

Malignancy	1.4%	1.4%	1.4%	1.4%	>0.99	0.0002	0.0002

Chronic kidney disease	2.8%	2.8%	2.8%	2.8%	>0.99	0.0002	0.0002

COPD or asthma	6.4%	6.5%	6.4%	6.4%	>0.99	0.0001	0.0001

Antiplatelet therapy	99.5%	99.5%	99.5%	99.5%	0.95	0.0002	0.0002

Lipid-lowering therapy	94.9%	94.9%	94.8%	94.9%	>0.99	0.0005	0.0005

RAAS inhibitors	78.1%	78.1%	78.1%	78.1%	>0.99	0.0003	0.0003

β-Blockers	83.3%	83.3%	83.3%	83.3%	>0.99	0.0001	0.0001

Anti-ischemic therapy	15.7%	15.7%	15.7%	15.7%	>0.99	0.0002	0.0002

Revascularization	95.5%	95.6%	95.5%	95.5%	>0.99	0.0005	0.0005

Number of diagnoses*	3 (2–4)	3 (2–4)	3 (2–4)	3 (2–4)	0.14	0.0012	0.0402

Number of procedures*	17 (12–20)	17 (12–20)	17 (12–20)	17 (13–20)	0.79	0.0004	0.0465

Length of stay ≥5 days	41.3%	41.3%	41.3%	41.4%	>0.99	0.0008	0.0008

DRG intensity*	3.4 (3.0–3.8)	3.4 (3.0–3.8)	3.4 (3.0–3.8)	3.6 (3.2–3.8)	<0.0001	0.0047	0.1610

University hospital	70.0%	70.0%	70.0%	70.0%	>0.99	0.0003	0.0003

Hospital providing CR**	93.1%	93.1%	93.1%	93.1%	>0.99	0.0001	0.0001

Socioeconomic status							

High	28.6%	28.7%	28.5%	28.5%	>0.99	0.0013	0.0013

Middle-High	18.6%	18.6%	18.6%	18.6%		0.0015	0.0015

Middle-Low	16.8%	16.8%	16.8%	16.8%		0.0004	0.0004

Low	36.0%	35.9%	36.1%	36.1%		0.0002	0.0002

Unadjusted sample size		7996	2281	1538			

Adjusted effective sample size		3388	1208	1538			

Weighted sample size		1534	1537	1538			


CR, cardiac rehabilitation; STEMI, ST elevation myocardial infarction (vs. non-ST elevation myocardial infarction); COPD, chronic obstructive pulmonary disease; RAAS, renin-angiotensin system inhibitors; CABG, coronary artery bypass grafting; PCI, percutaneous coronary intervention. *DRG – disease-related group; reflecting the intensity of hospital episode, including case mix, diagnoses, procedures, and complications. Total number of coded diagnoses and procedures, capturing all diagnoses and procedures coded during the hospital episode, including diagnoses and procedures not captured by DRG intensity. **Hospital providing affiliated cardiac rehabilitation center.

**Figure 4:**
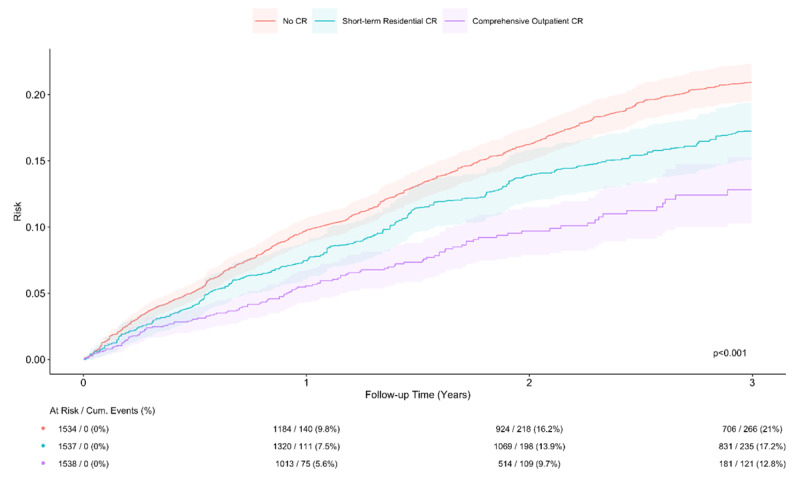
Kaplan–Meier freedom from all-cause mortality and cardiovascular hospitalization.

During a median follow-up time of 799 (IQR 354–1098) days, 2787 patients experienced a composite outcome (121 per 1000 patient-years), 1737 were hospitalized because of cardiovascular causes (71 per 1000 patient-years), and 1457 died (57 per 1000 patient-years). Survival curves (adjusted with inverse probability weights) for composite all-cause mortality and cardiovascular hospitalizations are depicted in Figure 4 (and in Supplementary Figure 2A–C for secondary outcomes – all-cause mortality and cardiovascular hospitalizations separately). Estimated event rates at 3-year follow-up were 12.8% (10.3%–15.3%), 17.2% (95% CI 15.1%–19.4%), and 21.0% (95% CI 19.5%–22.4%) for no CR, short-term residential, and no CR, respectively (*p* < 0.001).

Participation in either CR modality was associated with a significant risk reduction for the composite outcome of all-cause mortality and cardiovascular hospitalization: HR 0.58 (95% CI 0.47–0.7, *p* < 0.001) for comprehensive outpatient CR, and HR 0.79 (95% CI 0.68–0.93, *p* = 0.003) for short-term residential CR. All-cause mortality risk was also reduced in both groups: HR 0.56 (95% CI 0.38–0.83, *p* = 0.004) for comprehensive outpatient CR and HR 0.59 (95% CI 0.45–0.77, *p* < 0.001) for short-term residential CR. Comprehensive outpatient CR was also associated with a significant risk reduction for cardiovascular hospitalizations (HR 0.60, 95% CI 0.48–0.74, *p* < 0.001), whereas short-term residential CR was not (HR 0.88, 95% CI 0.73–1.04, *p* = 0.14). See Supplementary Table 6.

Sensitivity analyses (Supplementary Table 7) yielded comparable estimates, with HRs for composite events ranging from 0.50 (95% CI 0.42–0.60, *p* < 0.001) for comprehensive outpatient CR and 0.75 (95% CI 0.65–0.86, *p* < 0.001) for short-term residential CR, respectively, using the 90-day landmark, to 0.60 (95% CI 0.49–0.74, *p* < 0.001) for comprehensive outpatient CR and 0.84 (95% 0.70–0.99, *p* = 0.046) for short-term residential CR, respectively, using generalized boosted model-derived propensity scores.

Participation in comprehensive outpatient CR – but not in short-term residential CR – was also associated with significantly higher retention of all major secondary preventive medications at 12 months, with ORs ranging from 1.35 (1.21–1.52) for ACEi/ARBs to 3.12 (2.84–3.44) for lipid-lowering therapy. The impact of either CR on ER visits at 12 months was not significant (Supplementary Table 8).

### Cost-effectiveness analysis

Incremental costs (weighted mean) were €1695 (95% CI 1458–1943) for comprehensive outpatient CR and €1593 (1296–1892) for short-term residential CR, respectively, whereas restricted mean survival gains were 0.264 years (95% CI 0.239–0.289) for comprehensive CR and 0.216 years (95% CI 0.191–0.241) for short-term residential CR, respectively. Compared to no CR, the cost-effectiveness ratio per year-life gained was €6421 for comprehensive outpatient CR (95% CI 5401–7577) and €7381 (95% CI 5821–9112) for short-term residential CR (Supplementary Figure 3A and B).

## Discussion

Our analysis in one of the largest nationally representative patient populations provides real-life (externally valid/generalizable) evidence of the effectiveness of CR and is the first to compare comprehensive outpatient (as recommended by the 2021 ESC prevention guidelines) (2) and short-term residential (which remains popular in German-speaking countries) (10). After a dedicated comprehensive outpatient CR was introduced in 2017, nation-wide CR participation significantly increased both in level and in trend. More importantly, participation in either CR was associated with improved cardiovascular outcomes, but comprehensive outpatient CR yielded superior risk reductions, primarily through reduced cardiovascular hospitalizations.

### Participation in cardiac rehabilitation

Seven regional centers providing comprehensive outpatient CR were established in Slovenia between 2017 and 2020. CR density substantially increased, as defined by a reduction in incident ischemic heart disease per CR center (from 5568 to 1392) and per CR spot (from 37 to 9), thereby upgrading Slovenia, *ceteris paribus*, from a middle- to an upper-tertile country in terms of CR density (13). CR participation rates after myocardial infarction markedly increased – from 25% before the introduction of comprehensive outpatient CR programs in 2016 to 40% by 2020. The increase is remarkably similar to increases in CR participation over the 2010–2017 period in the Netherlands; the latter, however, could not be associated with a specific intervention (16). Conversely, using interrupted time series analysis – a robust quasi-experimental method for causal estimation when randomization is not feasible (non-randomized roll-out) or possible (COVID-19 pandemic) – we have shown that expansions of CR delivery (by establishing additional CR programs) yield a significant increase in both level (by ~10%) and in trend (~0.4% per month) of CR participation.

The increase in CR participation was promising, but still below recommended targets (32) or CR uptakes reported in studies of academic-affiliated hospitals (11). On the one hand, roughly half of the hospitals in the country managed to establish a functional CR program before 2020, suggesting room for further expansion. In addition, the increasing trend in CR participation was thwarted by the COVID-19 pandemic. A disruption in healthcare provision, including the provision of CR, due to the COVID-19 pandemic has been extensively reported (33,34). We have previously shown that the pandemic reduced myocardial infarction hospitalization rates and post-discharge secondary prevention uptake in Slovenia (35). Combined with the underutilization of CR, these disruptions in evidence-based processes of cardiovascular care may likely translate into unfavorable outcomes. Such analysis, however, may prove challenging because of fast-shifting healthcare trajectories during the pandemic (including diversion of healthcare resources, public health guidance, and evolving knowledge of the disease). For instance, while our analysis was limited to the earlier stages of the COVID-19 pandemic (up to 2021), a non-significant uptick (~0.2% per month) after the initial disruption may reflect the adaptation and recovery trajectory of CR programs. Of note, patients undergoing CR during the height of the pandemic reported that acute myocardial infarction affected their lives more than the pandemic itself (36).

On the other hand, CR remained underutilized in specific patient populations, such as women, older, and high-risk patients. Female sex, older age, and non-cardiac co-morbidities were also major predictors of both CR non-participation and CR non-completion at the individual patient level, which is consistent with findings from previous studies (37). In particular, sex differences in CR utilization have long been a major topic of interest (38); in our analysis, men were 15%–20% more likely than women to enroll and to complete either CR program. Commonly identified barriers to CR utilization in women include suboptimal referral and lack of CR awareness, older age and multiple co-morbidities at the time of coronary event, lack of social support (e.g., more caregiver responsibilities), lower baseline levels of exercise capacity and activity, unfavorable perceptions of exercise training, programs predominantly catering to, and attended by, men (39,40). Conversely, CR referral, increasing CR awareness and designing more flexible programs tailored for women have been proposed as possible solutions, which should guide further improvement of CR programs (38).

### Effectiveness of cardiac rehabilitation

Our analysis is the first to report on the comparative effectiveness of two distinctive CR modalities (comprehensive outpatient and short-term residential) in a nationally representative cohort. Participation in either CR was associated with improved event-free survival: the risk of the composite outcome (all-cause mortality and cardiovascular hospitalizations) was reduced by 42% and 21% with comprehensive outpatient and short-term residential CR, respectively. Based on estimated absolute risk reductions, the number of patients that should undergo CR to prevent a major event over 1 year would be 26 for comprehensive outpatient and 52 for short-term CR, respectively. The difference in risk reductions between the two CR modalities was primarily driven by cardiovascular hospitalizations, which were significantly reduced only with comprehensive outpatient CR (by 40%). Conversely, risk reductions for all-cause mortality were comparable, yet still larger with comprehensive outpatient CR (44% vs. 41% with short-term CR). This is reflected in favorable cost-effectiveness ratio per year-life gained (€6421 for comprehensive outpatient CR vs. €7381 for short-term CR), which is way above the willingness to pay threshold for any effective intervention.

Survival benefits in our analysis are closely aligned with those from other observational studies of CR participation in large community-based cohorts (16,18,21). It is noteworthy, however, that observed reductions in all-cause mortality are larger than those reported in meta-analyses of contemporary randomized trials. The latest Cochrane systematic review of 85 trials randomizing 23,430 patients with coronary artery disease reiterated that exercise-based CR reduces several outcomes (i.e., cardiovascular mortality by 24%, myocardial infarction by 18%, and all-cause hospitalization by 23% over a median follow-up of 12 months) – but not all-cause mortality (41). The prevailing explanation points to secular trends of improved care and falling mortality in patients with coronary artery disease, which have rendered all-cause mortality benefits difficult to detect in recent randomized trials of CR (16,21,41,42,43). In this regard, observational studies may provide complementary real-life estimation of CR survival benefits in unselected populations and uncontrolled settings (such as our nationally representative population of patients with myocardial infarction). Moreover, CR programs in randomized trials represent a wide range of interventions, from home-based programs to predominantly exercise-focused CR (41). Conversely, the primary focus of our analysis was comprehensive center-based CR programs. These two major determinants of our comprehensive outpatient CR – comprehensive content (44) and center-based delivery (6) – have been previously associated with improved outcomes. Comprehensive programs seem to have an upper hand in risk reductions: a systematic review of 18 trials randomizing 7691 patients to cardiovascular prevention and rehabilitation programs (44) identified two major program characteristics associated with reduced all-cause mortality – that is, the addressing of six or more risk factors (by 37%) and the provision of cardioprotective medications (by 65%). Both interventions are core components of our comprehensive outpatient CR programs and thus possible conveyors of larger risk reduction.

Our results also provide a perspective on short-term residential CR. Despite the widespread provision of short-term (2–4 weeks) residential exercise-based programs, especially in German-speaking countries (10), the available evidence on the prognostic effectiveness of such CR modalities is fairly limited (8,9). In our analysis, comprehensive CR was superior to short-term CR – both in terms of larger mortality risk reductions and in terms of significant risk reductions for cardiovascular hospitalization. This finding is somewhat expected, as comprehensive outpatient CR – comprising 36 sessions over 3 months – provides the necessary structure and duration for adequate delivery of evidence-based core interventions. While short-term CR improves functional capacity, risk factors, and quality of life, its provision over a relatively short period of time may prove challenging to accommodate the recommended dose and volume of CR necessary for long-term improvements. For one, secondary preventive medication retention at 12 months was significantly higher in the comprehensive CR group and thus one likely driver of the larger risk reduction. Nonetheless, participation in short-term residential CR remains superior to no CR participation at all and may thus provide a valid CR option when outpatient participation is not feasible or possible.

### Strengths and limitations

Our analysis provides important insights into the comparative effectiveness of two different CR modalities (i.e., comprehensive outpatient or short-term residential) in a nationally representative cohort – both in terms of quality of care (i.e., increased CR participation) and in terms of effectiveness. Nonetheless, our study has several limitations.

Firstly, this was an observational study relying on administrative healthcare datasets. Observational methodology may provide valid evidence on the association – but not causation – between CR participation and improved clinical outcomes. Causal inference from propensity score-adjustment assumes no unmeasured confounders, whereas our analysis could not capture several known determinants of referral to, adherence with, and outcomes after CR, including – but not limited to – smoking (45), exercise capacity (46), diet (47), or individual patient-level socio-economic determinants (48). In addition, groups were adjusted to reflect CR participants, which were younger, more often men, and had fewer co-morbidities (average treatment on the treated); the estimated effectiveness of CR may therefore not be generalizable to all patients after myocardial infarction.

Secondly, the outcomes analysis was limited to all-cause mortality and cardiovascular hospital readmissions, whereas CR provides broader patient-centered benefits, such as improvement in exercise capacity and functional status, mental health and well-being, long-term adherence to lifestyle changes and medication, patient experience, and health-related quality of life (1,41,49). Lack of data on health-related quality of life also limits our analysis to cost-effectiveness (life-years gained) instead of cost-utility (quality-adjusted life-years gained).

Thirdly, the analysis was limited to patients after myocardial infarction. Indications for CR encompass a wider range of patients – priority evidence-based indications to reduce cardiovascular events include any form of coronary artery disease (not only myocardial infarction) as well as heart failure (especially with reduced ejection fraction), but CR may also be considered for functional improvement in patients with other cardiovascular conditions, such as peripheral artery disease, congenital heart disease or atrial fibrillation (1,41). While patients with indications other than myocardial infarction were eligible for CR participation in Slovenia on a case-by-case basis, an expansion of systematic referral to other diagnoses provides an opportunity for further improvement.

## Conclusions

The present study reports on the implementation of comprehensive CR programs at roughly half of the regional hospitals in Slovenia, focusing on nation-wide CR participation and patient-level outcomes. Our findings suggest that establishing regional comprehensive outpatient CR centers yield a significant and meaningful increase in nationwide CR participation (which was, however, partially reversed by the COVID-19 pandemic during the time frame of our study). More importantly, our analysis of a real-life nation-wide patient population after myocardial infarction confirms that participation in CR – especially in comprehensive outpatient CR – yields improved patient-level clinical outcomes. While limited in scale (given the relatively small population of Slovenia) and scope (patients after myocardial infarction), our results should encourage decision-makers to endorse and/or expand implementation of comprehensive CR programs – both as a means to increase CR participation and as an effective intervention to improve outcomes in patients with cardiovascular diseases.

## Data Accessibility Statement

The data underlying this article were provided by *Healthcare Insurance Institute of Slovenia*; restrictions apply to the availability of the data, which were used under license for this study and can only be shared on request to the corresponding author with permission of *Healthcare Insurance Institute of Slovenia*.

## Additional File

The additional file for this article can be found as follows:

10.5334/gh.1470.s1 Supplementary file. Tables S1 to S8 and Figures S1 to S3.
