# Supplementary material for: Uptake and Effectiveness of Outpatient vs. Residential Cardiac Rehabilitation After Myocardial Infarction: A Nationwide Analysis
Source: Glob Heart. 2025 Sep 12;20(1):80. doi: 10.5334/gh.1470 (PMC12427614; doi:10.5334/gh.1470)
Supplement: Supplementary file. — Tables S1 to S8 and Figures S1 to S3. [file gh-20-1-1470-s1.pdf]

Supplementary Tables

Supplementary Table 1: Content overview for short-term residential and comprehensive outpatient cardiac rehabilitation

Supplementary Table 2: Subgroup and sensitivity analyses of interrupted time series for nation-wide cardiac rehabilitation uptake

Supplementary Table 3: Baseline characteristics with pairwise comparison between non-participants, short-term residential cardiac rehabilitation participants, and comprehensive outpatient cardiac rehabilitation participants

Supplementary Table 4: Predictors of participation to any cardiac rehabilitation (CR), short-term residential CR, and comprehensive outpatient CR

Supplementary Table 5: Predictors of completion of comprehensive outpatient cardiac rehabilitation (≥36 sessions) and short-term residential cardiac rehabilitation (≥14 days).

Supplementary Table 6: Double-robust Cox proportional hazards regression models for composite outcomes, all-cause mortality, and cardiovascular (CV) hospitalisations.

Supplementary Table 7: Sensitivity analysis for Cox proportional hazards regression models for composite event: 90-day landmark timeframe; generalised boosted model and multinomial regression model-derived propensity scores for weighting; standard (unweighted) multivariate and univariate Cox proportional hazard regression

Supplementary Table 8: All-cause mortality, cardiovascular hospitalisations, emergency room visits, and key secondary preventive medications at 12 months (weighted / pseudo-cohort analysis)

Supplemental Figures

Supplemental Figure 1: Covariate balance diagnostics (MPS — multinomial logistic regression, GBM — general boosted model, CBPS — covariate balancing propensity score).

Supplemental Figure 2: Kaplan-Meier freedom from (A) all-cause mortality and cardiovascular hospitalisation, (B) all-cause mortality, (C) cardiovascular hospitalisation.

Supplemental Figure 3: Cost-effectiveness analysis — cost-effectiveness plane (A) and cost-effectiveness acceptability curve

Supplementary Table 1: Content overview for short-term residential and comprehensive outpatient cardiac rehabilitation

	**Short-Term Residential**	**Comprehensive Outpatient**
**Setting**	Residential	Outpatient
**Location**	2 accredited residential centres (patient stays on-site)	5 regional hospitals, 2 university hospitals, 1 residential centre (upgraded to provide both programs)
**Duration**	2 weeks (residential)	3 months (outpatient)
**Location**	2 accredited residential centres (patient stays on-site)	Outpatient clinics
**Sessions**	24 sessions (twice daily)	36 sessions (2–3 times/week)
**Typical Exercise Content***	30 min moderate-intensity aerobic (60–80% of peak heart rate) + low-intensity resistance + relaxation, twice daily, 6 days/week	60 min moderate-intensity aerobic (60–80% of peak heart rate) + low-intensity resistance + relaxation, 2–3 times per week
**Initial Assessment**	Cardiovascular assessment	Cardiovascular assessment, initial exercise test, laboratory analyses
**Final Assessment**	Exercise test, echocardiography, long-term management recommendations	Exercise test, laboratory analyses, echocardiography, structured transition to long-term care
**Medical Supervision**	Medical supervision	Cardiologist-led secondary prevention and medication management
**Lifestyle and Education**	Lifestyle, nutrition education, psychosocial support	Nurse-led patient education, nutritional counselling, health promotion (group + individual sessions)
**Psychosocial Support**	Included during residential stay	Mood disorder screening + access to mental health consultation
**Risk Factor Management**	Risk factor appraisal and recommendations for long-term control	Specialist management of blood pressure, lipids, diabetes; smoking cessation; weight management programmes
**Transition to Community Programmes**	General recommendations at discharge	Structured discharge and referral to ‘phase III’ community CR programmes (“Coronary Clubs”)

* Exercise type and intensity were prescribed individually, based on initial patient assessment.

Supplementary Table 2 a-e: Subgroup and sensitivity analyses of interrupted time series for nation-wide cardiac rehabilitation uptake

Supplementary Table 2a: *Sensitivity analysis for change in interruption*

Characteristic		Sensitivity Intervention			Sensitivity COVID-19		
		Beta	95% CI	p-value	Beta	95% CI	p-value
(Intercept)		27	24 to 30	<0·0001	28	25 to 30	<0·0001
(Month)		-0·12	-0·33 to 0·09	0·26	-0·14	-0·29 to 0·01	0·070
Intervention	Level (%)	7·3	3·0 to 12	0·0012	9·7	6·2 to 13	<0·0001
	Trend (% per month)	0·39	0·15 to 0·64	0·0025	0·41	0·22 to 0·60	<0·0001
COVID-19	Level (%)	-8·1	-20 to 3·8	0·18	6·9	-4·1 to 18	0·21
	Trend (% per month)	0·17	-1·1 to 1·4	0·79	-2·2	-4·0 to -0·33	0·022

Supplementary Table 2b: *Predefined subgroups - Type of myocardial infarction*

Characteristic		STEMI			NSTEMI		
		Beta	95% CI	p-value	Beta	95% CI	p-value
(Intercept)		36	32 to 40	<0·0001	20	17 to 24	<0·0001
(Month)		-0·10	-0·34 to 0·13	0·38	-0·16	-0·36 to 0·04	0·12
Intervention	Level (%)	11	5·6 to 17	0·0001	8·3	3·6 to 13	0·0008
	Trend (% per month)	0·39	0·08 to 0·69	0·014	0·43	0·17 to 0·69	0·0019
COVID-19	Level (%)	-3·8	-15 to 7·5	0·50	-12	-19 to -4·1	0·0035
	Trend (% per month)	-0·88	-2·9 to 1·2	0·40	0·67	-0·72 to 2·1	0·33

Supplementary Table 2c: *Predefined subgroups - Sex*

Characteristic		Men			Women		
		Beta	95% CI	p-value	Beta	95% CI	p-value
(Intercept)		30	27 to 32	<0·0001	23	15 to 30	<0·0001
(Month)		-0·15	-0·30 to 0·01	0·066	-0·09	-0·52 to 0·35	0·69
Intervention	Level (%)	14	11 to 18	<0·0001	0·19	-9·6 to 10	0·97
	Trend (% per month)	0·35	0·15 to 0·55	0·0010	0·48	-0·09 to 1·1	0·10
COVID-19	Level (%)	-7·3	-14 to -0·12	0·047	-6·8	-20 to 6·3	0·30
	Trend (% per month)	0·32	-1·0 to 1·6	0·63	-0·35	-2·8 to 2·1	0·77

Supplementary Table 2d: *Predefined subgroups - Age*

Characteristic		<=65 Years			>65 Years		
		**Beta**	**95% CI**	**p-value**	**Beta**	**95% CI**	**p-value**
(Intercept)		33	30 to 36	<0·0001	22	18 to 26	<0·0001
(Month)		-0·10	-0·28 to 0·07	0·25	-0·17	-0·40 to 0·06	0·14
**Intervention**	Level (%)	14	9·6 to 18	<0·0001	6·0	0·85 to 11	0·023
	Trend (% per month)	0·59	0·36 to 0·82	<0·0001	0·25	-0·05 to 0·54	0·10
**COVID-19**	Level (%)	-14	-22 to -5·4	0·0021	-3·4	-12 to 5·1	0·42
	Trend (% per month)	0·09	-1·5 to 1·7	0·91	0·13	-1·4 to 1·7	0·87

Supplementary Table 2e: *Predefined subgroups - Hospital type*

Characteristic		University Hospital			Regional Hospital		
		**Beta**	**95% CI**	**p-value**	**Beta**	**95% CI**	**p-value**
(Intercept)		30	25 to 34	<0·0001	23	19 to 27	<0·0001
(Month)		-0·19	-0·46 to 0·07	0·15	-0·07	-0·29 to 0·15	0·52
**Intervention**	Level (%)	15	9·3 to 22	<0·0001	0·10	-5·0 to 5·2	0·97
	Trend (% per month)	0·39	0·05 to 0·74	0·026	0·65	0·36 to 0·93	<0·0001
**COVID-19**	Level (%)	-12	-22 to -1·2	0·030	3·3	-6·1 to 13	0·49
	Trend (% per month)	0·38	-1·6 to 2·3	0·69	-1·4	-3·2 to 0·27	0·10

Supplementary Table 3: Baseline characteristics with pairwise comparison between non-participants, short-term residential cardiac rehabilitation participants, and comprehensive outpatient cardiac rehabilitation participants

Characteristic	Overall	No CR	Short-term	Compr.	Overall p-value	Pairwise comparisons (Bonferroni adjusted p)		
	N = 11 815	N = 7 996	N = 2 281	N = 1 538		*Short-term vs. No CR*	*Comp. vs. No CR*	*Short-term vs. No CR*
Age	66 (57-77)	69 (59-79)	63 (55-71)	58 (52-66)	<0·0001	<0.0001	<0.0001	<0.0001
Sex (male)	7 882 (67%)	5 030 (63%)	1 676 (73%)	1 176 (76%)	<0·0001	<0.0001	<0.0001	0.1122
STEMI	5 431 (46%)	3 112 (39%)	1 410 (62%)	909 (59%)	<0·0001	<0.0001	<0.0001	0.2771
Diabetes	2 631 (22%)	1 827 (23%)	564 (25%)	240 (16%)	<0·0001	0.1839	<0.0001	p<0.0001
Arterial Hypertension	7 245 (61%)	5 030 (63%)	1 384 (61%)	831 (54%)	<0·0001	0.1569	<0.0001	0.0001
Atrial Fibrillation	1 481 (13%)	1 147 (14%)	260 (11%)	74 (4·8%)	<0·0001	0.0009	<0.0001	p<0.0001
Heart Failure	2 094 (18%)	1 366 (17%)	539 (24%)	189 (12%)	<0·0001	<0.0001	<0.0001	p<0.0001
Depression	811 (6·9%)	585 (7·3%)	147 (6·4%)	79 (5·1%)	0·0056	0.4603	0.0063	0.2790
Dementia	264 (2·2%)	258 (3·2%)	5 (0·2%)	1 (<0·1%)	<0·0001	<0.0001	<0.0001	0.7146
Malignancy	358 (3·0%)	285 (3·6%)	51 (2·2%)	22 (1·4%)	<0·0001	0.0050	<0.0001	0.2240
Chronic Kidney Disease	966 (8·2%)	768 (9·6%)	155 (6·8%)	43 (2·8%)	<0·0001	0.0001	<0.0001	<0.0001
COPD or Asthma	1 038 (8·8%)	748 (9·4%)	191 (8·4%)	99 (6·4%)	0·0008	0.4543	0.0007	0.0802
Antiplatelet Therapy	11 212 (95%)	7 493 (94%)	2 189 (96%)	1 530 (99%)	<0·0001	0.0001	<0.0001	<0.0001
Lipid-Lowering Therapy	9 727 (82%)	6 250 (78%)	2 018 (88%)	1 459 (95%)	<0·0001	<0.0001	<0.0001	<0.0001
RAAS Inhibitors	8 544 (72%)	5 629 (70%)	1 714 (75%)	1 201 (78%)	<0·0001	<0.0001	<0.0001	0.1071
Beta Blockers	8 834 (75%)	5 682 (71%)	1 871 (82%)	1 281 (83%)	<0·0001	<0.0001	<0.0001	0.9385
Antiischemic Therapy	2 381 (20%)	1 660 (21%)	479 (21%)	242 (16%)	<0·0001	1.0000	<0.0001	0.0001
Revascularisation								
CABG	252 (2·1%)	26 (0·3%)	222 (9·7%)	4 (0·3%)				
none	1 496 (13%)	1 265 (16%)	162 (7·1%)	69 (4·5%)	<0.0001	<0.0001	p<0.0001	0.0027
PCI	10 067 (85%)	6 705 (84%)	1 897 (83%)	1 465 (95%)				
Number of Diagnoses*	4 (2-5)	4 (2-5)	4 (3-6)	3 (2-4)	<0·0001	<0.0001	<0.0001	<0.0001
Number of Procedures*	15 (9-19)	13 (7-18)	16 (10-20)	17 (13-20)	<0·0001	<0.0001	<0.0001	0.1131
Length of Stay ≥5 days	6 242 (53%)	4 098 (51%)	1 508 (66%)	636 (41%)	<0·0001	<0.0001	<0.0001	<0.0001
DRG Intensity*	3·4 (2·4-3·8)	3·2 (2·3-3·8)	3·4 (2·7-4·2)	3·6 (3·2-3·8)	<0·0001	<0.0001	0.001	
University Hospital	6 895 (58%)	4 501 (56%)	1 318 (58%)	1 076 (70%)	<0·0001	0.6151	<0.0001	<0.0001
Hospital with CR Centre**	9 986 (85%)	6 710 (84%)	1 844 (81%)	1 432 (93%)	<0·0001	0.0016	<0.0001	<0.0001
Socio-Economic Status								
High	2 555 (22%)	1 706 (21%)	410 (18%)	439 (29%)				
Middle-High	2 706 (23%)	1 961 (25%)	459 (20%)	286 (19%)	<0·0001	<0.0001	<0.0001	<0.0001
Middle-Low	2 545 (22%)	1 761 (22%)	526 (23%)	258 (17%)				
Low	4 009 (34%)	2 568 (32%)	886 (39%)	555 (36%)				

CR – cardiac rehabilitation
STEMI — ST elevation myocardial infarction (vs. Non-ST elevation myocardial infarction)
COPD — chronic obstructive pulmonary disease
RAAS — renin-angiotensin system inhibitors
CABG – coronary artery bypass grafting
PCI – percutaneous coronary intervention
** Hospital providing affiliated cardiac rehabilitation center*
** DRG – disease related group; reflecting the intensity of hospital episode, including case mix, diagnoses, procedures, and complications. Total number of coded diagnoses and procedures, capturing all diagnoses and procedures coded during the hospital episode, including diagnoses and procedures not captured by DRG intensity*

Supplementary Table 4: Predictors of participation to any cardiac rehabilitation (CR), *short-term residential CR, and comprehensive outpatient CR*

	Any CR			Comprehensive outpatient CR			Short-term residential CR		
**Characteristic**	**OR[1]**	**95% CI[1]**	**p-value**	**OR[1]**	**95% CI[1]**	**p-value**	**OR[1]**	**95% CI[1]**	**p-value**
Age (years)	0·96	0·95 to 0·96	<0·0001	0·94	0·94 to 0·95	<0·0001	0·97	0·96 to 0·97	<0·0001
Sex (male)	1·21	1·10 to 1·33	0·0001	1·19	1·03 to 1·39	0·022	1·22	1·08 to 1·37	0·0011
STEMI vs. NSTEMI	1·99	1·81 to 2·18	<0·0001	1·60	1·39 to 1·84	<0·0001	2·33	2·09 to 2·61	<0·0001
Diabetes Mellitus	1·02	0·91 to 1·14	0·71	0·80	0·67 to 0·95	0·014	1·13	0·99 to 1·28	0·065
Arterial Hypertension	1·01	0·91 to 1·11	0·87	0·95	0·81 to 1·10	0·48	1·04	0·92 to 1·17	0·55
Atrial Fibrillation	0·83	0·71 to 0·97	0·019	0·58	0·44 to 0·77	0·0002	0·97	0·81 to 1·15	0·72
Heart Failure	1·31	1·15 to 1·48	<0·0001	0·88	0·71 to 1·08	0·21	1·55	1·35 to 1·78	<0·0001
Residual Ischemia	1·28	1·15 to 1·43	<0·0001	1·13	0·95 to 1·36	0·17	1·34	1·18 to 1·53	<0·0001
Depression	0·85	0·71 to 1·02	0·077	0·76	0·56 to 1·00	0·054	0·91	0·73 to 1·12	0·37
Dementia	0·12	0·04 to 0·25	<0·0001	0·08	0·00 to 0·38	0·014	0·12	0·04 to 0·27	<0·0001
Malignancy	0·61	0·45 to 0·81	0·0009	0·61	0·36 to 0·99	0·055	0·62	0·44 to 0·87	0·0068
Chronic Kidney Disease	0·79	0·65 to 0·96	0·020	0·66	0·45 to 0·94	0·026	0·82	0·66 to 1·02	0·073
COPD or Asthma	0·94	0·80 to 1·10	0·44	0·87	0·68 to 1·12	0·29	0·99	0·81 to 1·19	0·88
Antiplatelet Therapy	1·05	0·80 to 1·38	0·73	5·82	2·88 to 13·5	<0·0001	0·72	0·54 to 0·97	0·027
Lipid Lowering Therapy	1·55	1·33 to 1·82	<0·0001	1·90	1·45 to 2·52	<0·0001	1·43	1·19 to 1·72	0·0001
RAAS Inhibitor	1·04	0·93 to 1·16	0·50	1·10	0·94 to 1·30	0·24	1·03	0·91 to 1·18	0·64
Beta Blocker	1·29	1·15 to 1·45	<0·0001	1·34	1·13 to 1·59	0·0010	1·30	1·13 to 1·50	0·0002
Revascularisation									
- None	—	—		—	—		—	—	
- CABG	18·5	11·2 to 31·3	<0·0001	0·56	0·14 to 1·84	0·37	30·2	18·1 to 51·9	<0·0001
- PCI	1·20	1·00 to 1·44	0·055	1·19	0·86 to 1·67	0·31	1·28	1·04 to 1·59	0·024
Number of Diagnoses*	1·03	1·01 to 1·05	0·0089	1·00	0·97 to 1·04	0·86	1·04	1·02 to 1·07	0·0013
Number of Procedures*	1·02	1·01 to 1·03	<0·0001	1·02	1·01 to 1·04	0·0010	1·01	1·00 to 1·02	0·010
Length of Stay ≥5 days	1·32	1·20 to 1·45	<0·0001	1·09	0·95 to 1·26	0·22	1·58	1·41 to 1·78	<0·0001
DRG Intensity*	1·00	1·00 to 1·00	0·021	1·00	1·00 to 1·00	0·20	1·00	1·00 to 1·00	0·0016
University Hospital**	1·02	0·91 to 1·16	0·69	1·45	1·21 to 1·73	<0·0001	0·85	0·74 to 0·99	0·037
CR Providing Hospital**	0·95	0·82 to 1·10	0·52	2·06	1·58 to 2·69	<0·0001	0·71	0·60 to 0·83	<0·0001
Socio-Economic Status***									
- SES.L	1·13	1·03 to 1·25	0·0087	1·22	1·06 to 1·41	0·0054	1·04	0·92 to 1·16	0·56
- SES.Q	1·21	1·12 to 1·31	<0·0001	1·54	1·38 to 1·73	<0·0001	1·06	0·97 to 1·16	0·21
Calendar Year									
- 2015	—	—		—	—		—	—	
- 2016	0·94	0·80 to 1·09	0·41	1·35	0·73 to 2·56	0·35	0·92	0·79 to 1·08	0·31
- 2017	1·15	0·99 to 1·33	0·077	9·83	6·10 to 16·8	<0·0001	0·85	0·72 to 1·00	0·046
- 2018	1·83	1·58 to 2·12	<0·0001	34·2	21·5 to 57·9	<0·0001	0·87	0·74 to 1·04	0·12
- 2019	2·07	1·78 to 2·40	<0·0001	53·5	33·7 to 90·6	<0·0001	0·65	0·54 to 0·78	<0·0001
- 2020	1·64	1·41 to 1·92	<0·0001	47·7	30·0 to 80·8	<0·0001	0·38	0·31 to 0·47	<0·0001
Distance from Rehab Center									
<10 km	—	—		—	—		—	—	
10-19 km	0·72	0·61 to 0·84	<0·0001	0·57	0·44 to 0·72	<0·0001	0·81	0·67 to 0·98	0·032
20-29 km	0·64	0·53 to 0·77	<0·0001	0·49	0·37 to 0·65	<0·0001	0·75	0·60 to 0·93	0·0091
30-39 km	0·58	0·48 to 0·70	<0·0001	0·46	0·34 to 0·61	<0·0001	0·65	0·52 to 0·81	0·0002
>40 km	0·48	0·41 to 0·57	<0·0001	0·47	0·37 to 0·59	<0·0001	0·48	0·40 to 0·59	<0·0001

CR – cardiac rehabilitation
OR – odds ratio
CI – confidence interval
STEMI — ST elevation myocardial infarction (vs. Non-ST elevation myocardial infarction)
COPD — chronic obstructive pulmonary disease
RAAS — renin-angiotensin system inhibitors
CABG – coronary artery bypass grafting
PCI – percutaneous coronary intervention
** DRG – disease related group; reflecting the intensity of hospital episode, including case mix, diagnoses, procedures, and complications. Total number of coded diagnoses and procedures, capturing all diagnoses and procedures coded during the hospital episode, including diagnoses and procedures not captured by DRG intensity*
*** Hospital providing affiliated cardiac rehabilitation center*
**** Community of residence socio-economic status (SES), modelled as a linear and quadratic ordinal factor variable*

Supplementary Table 5: Predictors of completion of comprehensive outpatient cardiac rehabilitation (≥36 sessions) and short-term residential cardiac rehabilitation (≥14 days).

	Completion of Comprehensive CR			Completion of Residential CR		
**Characteristic**	**OR**[1]	**95% CI**[1]	**p-value**	**OR**[1]	**95% CI**[1]	**p-value**
Age (years)	0·97	0·96 to 0·97	<0·0001	0·95	0·95 to 0·96	<0·0001
Sex (male)	1·24	1·10 to 1·40	0·0005	1·17	0·96 to 1·43	0·13
STEMI vs. NSTEMI	2·22	1·98 to 2·48	<0·0001	1·43	1·19 to 1·72	0·0001
Diabetes Mellitus	1·11	0·97 to 1·26	0·13	0·75	0·59 to 0·95	0·021
Arterial Hypertension	1·07	0·94 to 1·20	0·30	0·99	0·81 to 1·20	0·91
Atrial Fibrillation	1·00	0·84 to 1·19	>0·99	0·56	0·37 to 0·82	0·0045
Heart Failure	1·53	1·32 to 1·76	<0·0001	0·83	0·63 to 1·09	0·19
Residual Ischemia	1·29	1·13 to 1·47	0·0001	1·09	0·86 to 1·36	0·49
Depression	0·91	0·73 to 1·12	0·38	0·83	0·56 to 1·19	0·32
Dementia	0·11	0·03 to 0·27	<0·0001	0·00	0·00 to 0·00	0·95
Malignancy	0·66	0·46 to 0·92	0·018	0·58	0·27 to 1·12	0·14
Chronic Kidney Disease	0·77	0·61 to 0·96	0·023	0·58	0·33 to 0·97	0·047
COPD or Asthma	0·95	0·78 to 1·16	0·64	0·91	0·64 to 1·27	0·59
Antiplatelet Therapy	0·70	0·52 to 0·95	0·019	3·81	1·61 to 11·3	0·0060
Lipid Lowering Therapy	1·42	1·18 to 1·71	0·0002	1·83	1·27 to 2·70	0·0017
RAAS Inhibitor	1·04	0·92 to 1·19	0·52	1·03	0·83 to 1·28	0·78
Beta Blocker	1·32	1·15 to 1·52	0·0001	1·16	0·93 to 1·46	0·19
Revascularisation						
- None	—	—		—	—	
- CABG	23·5	14·6 to 38·4	<0·0001	N/A*	N/A*	N/A*
- PCI	1·40	1·13 to 1·75	0·0028	1·32	0·86 to 2·06	0·21
Number of Diagnoses**	1·04	1·01 to 1·07	0·0028	1·02	0·97 to 1·07	0·51
Number of Procedures**	1·01	1·00 to 1·03	0·0089	1·02	1·00 to 1·04	0·11
Length of Stay ≥5 days	1·57	1·40 to 1·77	<0·0001	1·26	1·05 to 1·51	0·014
DRG Intensity**	1·00	1·00 to 1·00	0·019	1·00	1·00 to 1·00	0·18
University Hospital	0·84	0·73 to 0·98	0·026	1·57	1·23 to 2·01	0·0003
CR Providing Hospital***	0·71	0·60 to 0·84	<0·0001	0·97	0·69 to 1·35	0·84
Socio-Economic Status****						
- SES.L	1·03	0·92 to 1·16	0·61	1·07	0·90 to 1·29	0·44
- SES.Q	1·07	0·97 to 1·17	0·18	1·79	1·54 to 2·10	<0·0001
Calendar Year						
- 2015	—	—		—	—	
- 2016	0·93	0·80 to 1·09	0·40	0·75	0·30 to 1·79	0·52
- 2017	0·86	0·73 to 1·02	0·077	6·51	3·63 to 12·7	<0·0001
- 2018	0·88	0·74 to 1·05	0·16	24·9	14·3 to 47·7	<0·0001
- 2019	0·68	0·56 to 0·82	<0·0001	26·7	15·4 to 51·2	<0·0001
- 2020	0·39	0·31 to 0·48	<0·0001	11·8	6·70 to 22·8	<0·0001
Distance from Rehabilitation Center						
<10 km	—	—		—	—	
10-19 km	0·83	0·68 to 1·01	0·063	0·49	0·37 to 0·65	<0·0001
20-29 km	0·74	0·59 to 0·93	0·0082	0·42	0·30 to 0·59	<0·0001
30-39 km	0·65	0·51 to 0·81	0·0002	0·29	0·20 to 0·42	<0·0001
>40 km	0·49	0·40 to 0·60	<0·0001	0·26	0·20 to 0·36	<0·0001

CR – cardiac rehabilitation; completion was defined as ≥36 sessions for comprehensive outpatient CR and ≥14 days for short-term residential CR, respectively
OR – odds ratio
CI – confidence interval
STEMI — ST elevation myocardial infarction (vs. Non-ST elevation myocardial infarction)
COPD — chronic obstructive pulmonary disease
RAAS — renin-angiotensin system inhibitors
CABG – coronary artery bypass grafting
PCI – percutaneous coronary intervention
** All participants to short-term CR completed 14 days of stay; modelled for PCI/no-PCI only*
*** DRG – disease related group; reflecting the intensity of hospital episode, including case mix, diagnoses, procedures, and complications. Total number of coded diagnoses and procedures, capturing all diagnoses and procedures coded during the hospital episode, including diagnoses and procedures not captured by DRG intensity*
**** Hospital providing affiliated cardiac rehabilitation center*
***** Community of residence socio-economic status (SES), modelled as a linear and quadratic ordinal factor variable*

Supplementary Table 6: Double-robust Cox proportional hazards regression models for composite outcomes, all-cause mortality, and cardiovascular (CV) hospitalisations

	Composite			All-Cause Mortality			CV Hospitalisations		
	**HR**	**95% CI**	**p-value**	**HR**	**95% CI**	**p-value**	**HR**	**95% CI**	**p-value**
Comprehensive CR	0·58	0·47 to 0·70	<0·0001	0·56	0·38 to 0·83	0·0035	0·60	0·48 to 0·74	<0·0001
Short-term CR	0·79	0·68 to 0·93	0·0033	0·59	0·45 to 0·77	0·0001	0·88	0·73 to 1·04	0·14
Age	1·02	1·01 to 1·03	<0·0001	1·07	1·05 to 1·08	<0·0001	1·01	1·00 to 1·02	0·066
Sex (male)	1·15	0·98 to 1·36	0·088	1·35	1·08 to 1·69	0·0095	1·08	0·89 to 1·31	0·43
STEMI	1·02	0·88 to 1·19	0·77	0·93	0·72 to 1·19	0·54	1·03	0·86 to 1·22	0·78
Diabetes	1·26	1·06 to 1·49	0·0071	1·57	1·19 to 2·07	0·0014	1·19	0·98 to 1·46	0·083
Arterial Hypertension	0·84	0·72 to 0·99	0·039	0·85	0·65 to 1·11	0·23	0·86	0·72 to 1·04	0·13
Atrial Fibrillation	1·13	0·91 to 1·40	0·26	1·15	0·85 to 1·56	0·37	1·03	0·79 to 1·34	0·85
Heart Failure	1·22	1·03 to 1·44	0·021	1·44	1·12 to 1·85	0·0044	1·16	0·95 to 1·42	0·16
Residual Ischaemia	1·14	0·97 to 1·34	0·11	1·05	0·81 to 1·37	0·70	1·21	1·01 to 1·45	0·039
Depression	1·53	1·19 to 1·97	0·0008	1·29	0·91 to 1·84	0·15	1·51	1·13 to 2·03	0·0058
Dementia	2·22	0·66 to 7·41	0·20	3·09	0·86 to 11·1	0·083	0·20	0·08 to 0·47	0·0002
Malignancy	1·39	0·96 to 2·01	0·082	1·85	1·17 to 2·93	0·0082	1·07	0·63 to 1·81	0·81
Chronic Kidney Disease	1·08	0·84 to 1·38	0·56	1·29	0·93 to 1·81	0·13	0·93	0·67 to 1·28	0·66
COPD or Asthma	1·16	0·93 to 1·45	0·19	1·89	1·37 to 2·62	0·0001	1·03	0·78 to 1·36	0·82
Antiplatelet Therapy	1·31	0·85 to 2·03	0·22	0·90	0·52 to 1·57	0·72	1·59	0·97 to 2·63	0·069
Lipid-Lowering Therapy	0·83	0·67 to 1·03	0·085	0·81	0·59 to 1·09	0·16	0·90	0·69 to 1·18	0·45
RAAS Inhibitors	0·93	0·78 to 1·11	0·42	0·83	0·64 to 1·09	0·18	0·95	0·77 to 1·16	0·61
Beta Blockers	0·93	0·78 to 1·12	0·47	0·77	0·56 to 1·05	0·10	1·03	0·83 to 1·29	0·76
Revascularisation	0·98	0·73 to 1·30	0·87	0·51	0·35 to 0·75	0·0006	1·58	1·08 to 2·30	0·017
Number of Diagnoses*	1·07	1·04 to 1·11	<0·0001	1·12	1·06 to 1·18	<0·0001	1·05	1·01 to 1·09	0·024
Number of Procedures*	0·99	0·98 to 1·01	0·31	1·02	0·99 to 1·04	0·28	0·99	0·97 to 1·00	0·11
Length of Stay ≥5 days	1·16	1·00 to 1·36	0·051	1·15	0·87 to 1·53	0·32	1·17	0·98 to 1·39	0·085
DRG Intensity*	1·03	0·98 to 1·07	0·25	1·00	0·95 to 1·06	0·88	1·04	0·98 to 1·09	0·17
University Hospital	0·95	0·79 to 1·14	0·56	0·65	0·48 to 0·87	0·0045	1·03	0·83 to 1·27	0·81
Hospital with CR Centre**	1·04	0·82 to 1·32	0·72	1·31	0·90 to 1·91	0·15	0·93	0·70 to 1·22	0·58
Socioeconomic Status									
High	—	—		—	—		—	—	
Middle-High	1·07	0·87 to 1·33	0·52	1·16	0·81 to 1·64	0·42	1·02	0·79 to 1·31	0·89
Middle-Low	1·01	0·81 to 1·26	0·95	0·75	0·52 to 1·08	0·13	1·07	0·83 to 1·37	0·60
Low	0·90	0·74 to 1·09	0·29	0·86	0·62 to 1·19	0·36	0·90	0·72 to 1·13	0·37

CV – cardiovascular
HR – Hazard Ratio
CI – Confidence Interval
CR – Cardiac rehabilitation
STEMI — ST elevation myocardial infarction (vs. Non-ST elevation myocardial infarction)
COPD — chronic obstructive pulmonary disease
RAAS — renin-angiotensin system inhibitors
** DRG – disease related group; reflecting the intensity of hospital episode, including case mix, diagnoses, procedures, and complications. Total number of coded diagnoses and procedures, capturing all diagnoses and procedures coded during the hospital episode, including diagnoses and procedures not captured by DRG intensity*
*** Hospital providing affiliated cardiac rehabilitation center*

Supplementary Table 7: Sensitivity analysis for Cox proportional hazards regression models for composite event: 90-day landmark timeframe; generalised boosted model and multinomial regression model-derived propensity scores for weighting; standard (unweighted) multivariate and univariate Cox proportional hazard regression

Characteristic	90-day timeframe			GMB Propensity Score			Multinomial Regression PS			Adjusted Unweighted			Unadjusted Weighted		
	HR	95% CI	p-value	HR	95% CI	p-value	HR	95% CI	p-value	HR	95% CI	p-value	HR	95% CI	p-value
Comprehensive CR	0·50	0·42 to 0·60	<0·0001	0·60	0·49 to 0·74	<0·0001	0·58	0·48 to 0·70	<0·0001	0·58	0·48 to 0·70	<0·0001	0·57	0·47 to 0·62	<0·0001
Short-term CR	0·75	0·65 to 0·86	<0·0001	0·84	0·70 to 0·99	0·046	0·78	0·67 to 0·91	0·002	0·72	0·64 to 0·80	<0·0001	0·81	0·69 to 0·94	0·007
Age	1·02	1·01 to 1·03	<0·0001	1·02	1·01 to 1·03	<0·0001	1·02	1·01 to 1·03	<0·0001	1·03	1·02 to 1·03	<0·0001			
Sex (male)	1·13	0·97 to 1·31	0·12	1·24	1·04 to 1·47	0·014	1·17	0·99 to 1·38	0·066	1·15	1·06 to 1·25	0·0006			
STEMI	1·02	0·88 to 1·17	0·83	0·98	0·83 to 1·17	0·84	1·02	0·88 to 1·19	0·75	0·96	0·88 to 1·04	0·28			
Diabetes	1·29	1·11 to 1·50	0·0009	1·38	1·15 to 1·67	0·0005	1·29	1·10 to 1·52	0·0022	1·23	1·13 to 1·34	<0·0001			
Arterial Hypertension	0·82	0·70 to 0·95	0·0069	0·80	0·67 to 0·96	0·017	0·85	0·72 to 1·00	0·046	0·88	0·81 to 0·96	0·0037			
Atrial Fibrillation	1·09	0·90 to 1·32	0·38	1·03	0·81 to 1·30	0·83	1·11	0·89 to 1·38	0·35	1·04	0·95 to 1·15	0·39			
Heart Failure	1·24	1·07 to 1·45	0·0041	1·24	1·02 to 1·51	0·031	1·23	1·03 to 1·46	0·021	1·29	1·17 to 1·41	<0·0001			
Residual Ischaemia	1·14	0·99 to 1·32	0·078	1·20	1·00 to 1·44	0·055	1·12	0·96 to 1·32	0·15	1·14	1·05 to 1·25	0·0025			
Depression	1·45	1·15 to 1·82	0·0015	1·59	1·19 to 2·11	0·0015	1·54	1·18 to 2·01	0·0013	1·25	1·09 to 1·42	0·0009			
Dementia	1·84	0·61 to 5·59	0·28	1·62	0·63 to 4·18	0·32	2·25	0·66 to 7·72	0·20	1·24	1·04 to 1·47	0·017			
Malignancy	1·43	1·01 to 2·01	0·041	1·20	0·80 to 1·78	0·38	1·37	0·93 to 2·03	0·11	1·44	1·23 to 1·70	<0·0001			
Chronic Kidney Disease	1·10	0·88 to 1·37	0·39	1·06	0·81 to 1·39	0·67	1·06	0·82 to 1·37	0·67	1·29	1·15 to 1·44	<0·0001			
COPD or Asthma	1·13	0·92 to 1·39	0·23	1·15	0·92 to 1·44	0·23	1·16	0·93 to 1·45	0·19	1·20	1·07 to 1·35	0·0018			
Antiplatelet Therapy	1·24	0·84 to 1·84	0·28	1·23	0·85 to 1·79	0·28	1·27	0·83 to 1·96	0·27	1·12	0·95 to 1·31	0·17			
Lipid-Lowering Therapy	0·84	0·69 to 1·02	0·080	0·79	0·62 to 1·01	0·062	0·83	0·67 to 1·04	0·10	0·73	0·66 to 0·81	<0·0001			
RAAS Inhibitors	0·93	0·79 to 1·09	0·35	0·93	0·78 to 1·12	0·44	0·91	0·77 to 1·09	0·32	0·87	0·80 to 0·95	0·0015			
Beta Blockers	0·93	0·78 to 1·10	0·38	0·89	0·73 to 1·08	0·22	0·95	0·80 to 1·14	0·61	0·98	0·90 to 1·07	0·63			
Revascularisation	0·97	0·75 to 1·26	0·84	1·19	0·85 to 1·65	0·31	1·04	0·77 to 1·42	0·79	0·73	0·65 to 0·82	<0·0001			
Number of Diagnoses*	1·07	1·04 to 1·11	<0·0001	1·07	1·03 to 1·11	0·0003	1·07	1·03 to 1·11	0·0004	1·06	1·04 to 1·07	<0·0001			
Number of Procedures*	0·99	0·98 to 1·01	0·36	1·00	0·98 to 1·01	0·60	0·99	0·98 to 1·01	0·24	1·00	0·99 to 1·01	0·52			
Length of Stay ≥5 days	1·21	1·05 to 1·39	0·0075	1·29	1·09 to 1·54	0·0032	1·18	1·01 to 1·38	0·036	1·16	1·06 to 1·26	0·0008			
DRG Intensity*	1·01	0·97 to 1·05	0·60	1·03	0·99 to 1·07	0·22	1·03	0·97 to 1·09	0·29	1·02	1·00 to 1·04	0·11			
University Hospital	0·90	0·76 to 1·06	0·21	0·89	0·72 to 1·09	0·26	0·96	0·80 to 1·16	0·67	0·92	0·83 to 1·02	0·10			
Hospital w/ CR Centre**	1·11	0·90 to 1·38	0·32	1·08	0·83 to 1·40	0·56	1·02	0·80 to 1·30	0·87	1·11	0·98 to 1·25	0·091			
Socioeconomic Status															
High	—	—	—	—	—	—	—	—	—	—	—	—			
Middle-High	1·07	0·88 to 1·30	0·50	1·13	0·90 to 1·43	0·30	1·09	0·88 to 1·37	0·43	1·07	0·95 to 1·20	0·24			
Middle-Low	0·97	0·79 to 1·18	0·74	1·16	0·90 to 1·51	0·25	1·01	0·81 to 1·25	0·93	1·01	0·89 to 1·14	0·92			
Low	0·91	0·77 to 1·08	0·30	0·96	0·77 to 1·18	0·67	0·91	0·76 to 1·11	0·36	1·00	0·89 to 1·11	0·94			

CV – cardiovascular
HR – Hazard Ratio
CI – Confidence Interval
CR – Cardiac rehabilitation
STEMI — ST elevation myocardial infarction (vs. Non-ST elevation myocardial infarction)
COPD — chronic obstructive pulmonary disease
RAAS — renin-angiotensin system inhibitors
** DRG – disease related group; reflecting the intensity of hospital episode, including case mix, diagnoses, procedures, and complications. Total number of coded diagnoses and procedures, capturing all diagnoses and procedures coded during the hospital episode, including diagnoses and procedures not captured by DRG intensity*
*** Hospital providing affiliated cardiac rehabilitation center*

Supplementary Table 8: All-cause mortality, cardiovascular hospitalisations, emergency room visits, and key secondary preventive medications at 12 months (weighted / pseudo-cohort analysis)

**Characteristic**	**Overall**	**No CR**	**Short-term**	**Comprehensive**	**p-value**	**Adjusted OR for short-term CR**	**Adjusted OR for comprehensive CR**
	N = 4, 609	N = 1, 534	N = 1 ,537	N = 1, 538			
All-Cause Mortality	1·4%	2·4%	1·1%	0·8%	0·0001	0·52 (0·38 to 0·72)	0·40 (0·28 to 0·57)
CV Hospitalizations	5·9%	7·2%	6·3%	4·2%	0·0005	0·86 (0·72 to 1·03)	0·56 (0·46 to 0·68)
ER Visits	2·6%	2·2%	2·8%	2·8%	0·48	1·32 (0·98 to 1·79)	1·27 (0·94 to 1·72)
Intensive Antithrombotic Therapy	86%	84%	85%	88%	0·0018	1·08 (0·95 to 1·23)	1·41 (1·24 to 1·62)
Lipid-lowering Therapy	57%	50%	48%	74%	<0·0001	0·95 (0·87 to 1·04)	3·12 (2·82 to 3·44)
Beta Blocker	62%	59%	61%	65%	0·0023	1·09 (0·99 to 1·21)	1·37 (1·24 to 1·52)
ACE inhibitor/ARB	64%	63%	60%	68%	<0·0001	0·86 (0·77 to 0·95,)	1·35 (1·21 to 1·51)

CR – Cardiac rehabilitation
OR – adjusted odds ratio with 95% confidence interval
CV – cardiovascular
ER – Emergency room
ACE – Angiotensin Converting Enzyme
ARB – Angiotensin Receptor Blocker

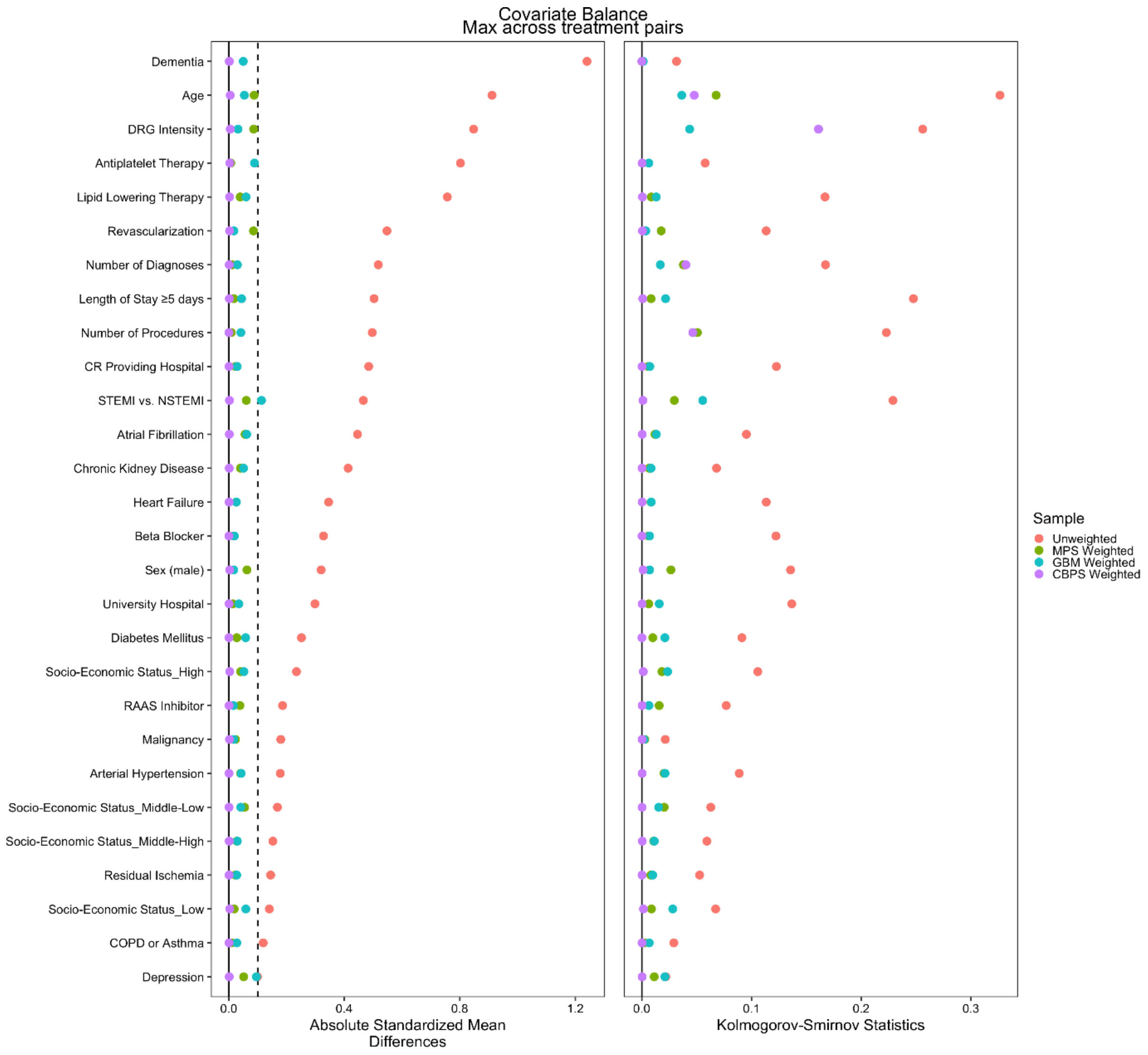

Supplementary Figure 1: Covariate balance diagnostics (MPS — multinomial logistic regression, GBM — general boosted model, CBPS — covariate balancing propensity score).

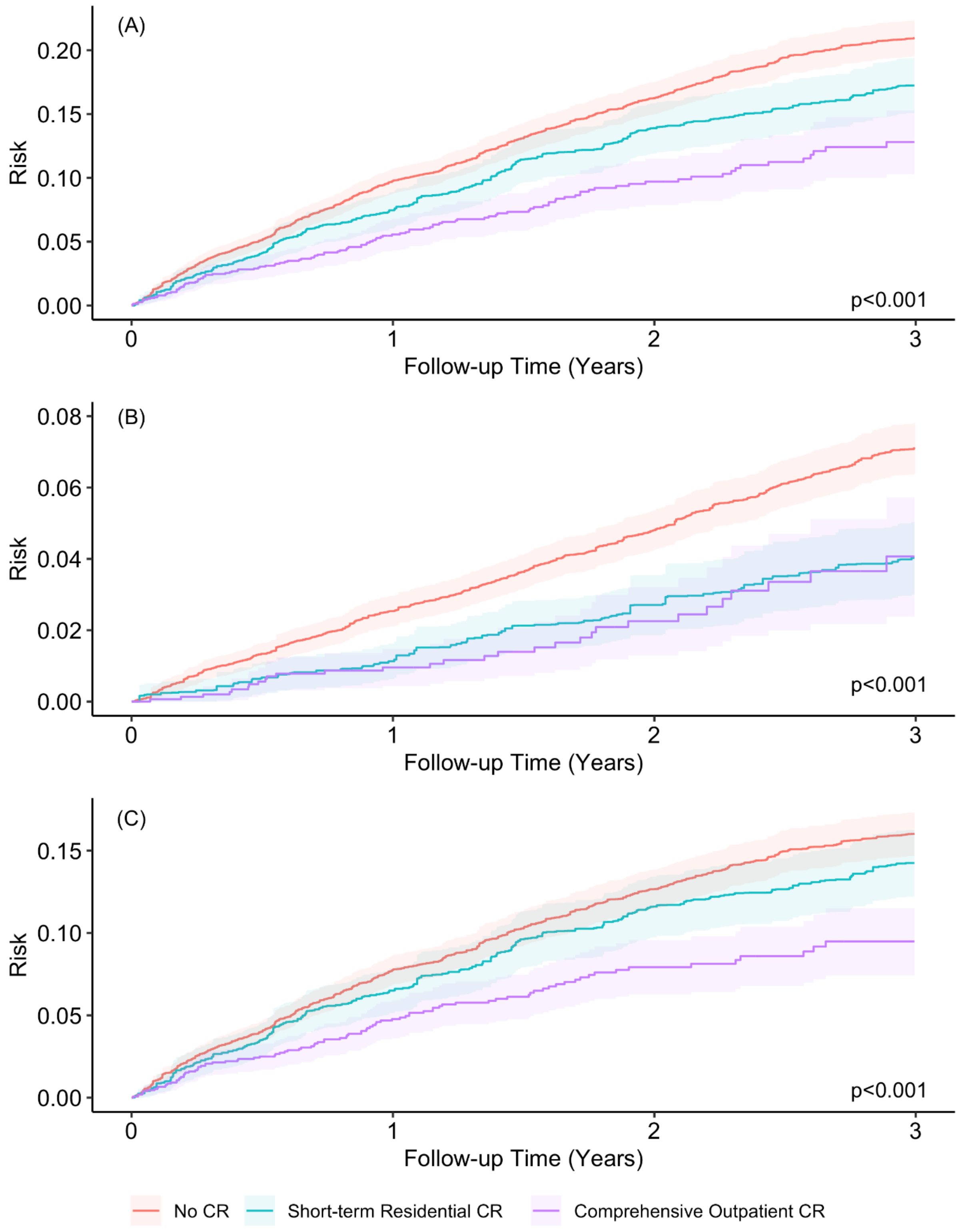

Supplementary Figure 2: Kaplan-Meier freedom from (A) all-cause mortality and cardiovascular hospitalisation, (B) all-cause mortality, (C) cardiovascular hospitalisation.

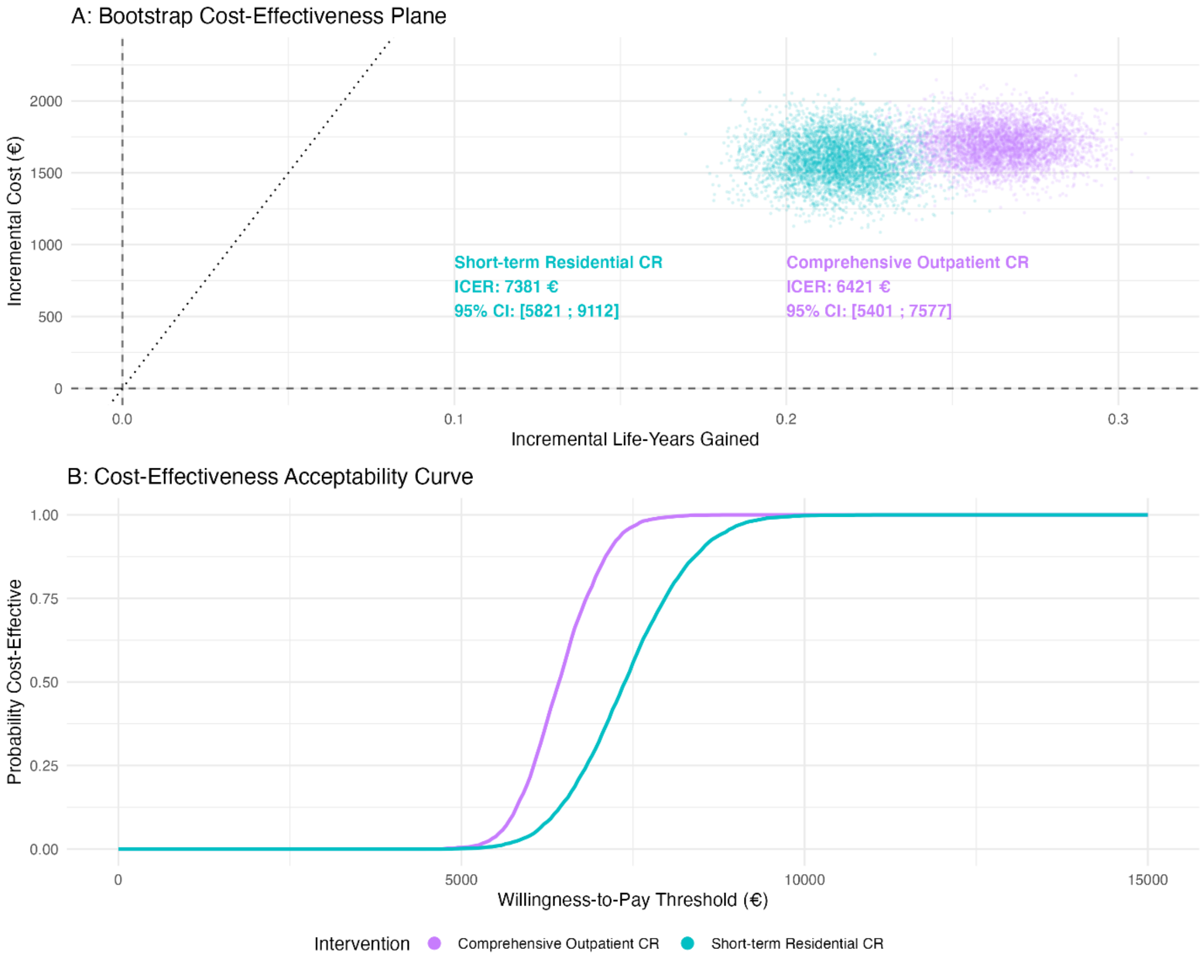

Supplemental Figure 3: Cost-effectiveness analysis — cost-effectiveness plane (A) and cost-effectiveness acceptability curve
